# Impact of Intensive Lifestyle Modification on Levels of Adipokines and Inflammatory Biomarkers in Metabolically Healthy Obese Women

**DOI:** 10.1155/2019/4165260

**Published:** 2019-04-09

**Authors:** Ricardo Gomez-Huelgas, Josefina Ruiz-Nava, Sonia Santamaria-Fernandez, Antonio Vargas-Candela, Ana Victoria Alarcon-Martin, Francisco J. Tinahones, M. Rosa Bernal-Lopez

**Affiliations:** ^1^Internal Medicine Department, Instituto de Investigación Biomedica de Malaga-IBIMA, Regional University Hospital of Malaga, Spain; ^2^CIBER Fisiopatologia de la Obesidad y la Nutricion, Instituto de Salud Carlos III, Madrid, Spain; ^3^University of Malaga, Spain; ^4^Endocrinology and Nutrition Department, Instituto de Investigación Biomedica de Malaga-IBIMA, University Hospital of Malaga (Virgen de la Victoria Hospital), Spain

## Abstract

**Background:**

For the metabolically healthy obese (MHO) subjects, it is unclear whether weight loss provides cardiometabolic benefits. Our objective was to evaluate whether changes in adipokine and inflammatory biomarker levels were related to lifestyle modification (with Mediterranean diet and physical exercise program).

**Methods:**

115 women (35-55 years) with BMI of 30-40 kg/m^2^ and ≤1 metabolic syndrome criteria were included. After a 2-year intervention, participants were classified by percent weight loss: Group 1, <5%; Group 2, ≥5%-<10%; and Group 3, ≥10%. Anthropometric data, inflammatory biomarker (IL-6, TNFa, and hsCRP) and adipokine levels (adiponectin and resistin), and lifestyle program adherence at baseline and 2 years were analyzed.

**Results:**

The final sample comprised 67 women. 23 (38.3%) lost <5%, 22 (36.7%) lost ≥5%-<10%, and 22 (36.7%) lost ≥10% of baseline weight. After 2 years, in Group 1, adiponectin, hsCRP, IL-6, and TNFa decreased (-1.2 ng/ml, *p* = 0.003; -2.1 mg/l, *p* = 0.003; -2.4 pg/ml, *p* < 0.001; and -2.4 pg/ml, *p* = 0.001, respectively) and resistin increased (+2.4 ng/ml, *p* < 0.001). In Group 2, hsCRP and IL-6 decreased (-2.0 mg/l, *p* = 0.009 and -2.6 pg/ml, *p* = 0.001) but TNFa increased (+0.2 pg/ml, *p* = 0.02). In Group 3, resistin increased (+3.5 ng/ml, *p* < 0.001) but hsCRP, IL-6, and TNFa decreased (-2.0 mg/l, *p* = 0.009; -2.5 pg/ml, *p* < 0.001; and -4.1 pg/ml, *p* < 0.001). Adiponectin, hsCRP, and physical exercise correlated significantly to subjects' dietary adherence.

**Conclusion:**

Weight loss reduces inflammatory biomarkers in the MHO but induces a deterioration in the adipokine profile, which does not improve with diet and exercise intervention. These findings allow us to clarify mechanisms behind inflammation and metabolic disorder genesis so as to prevent development of obesity-associated comorbidities.

## 1. Introduction

Obesity is a heterogeneous disease. The risk of developing complications associated with obesity, such as type 2 diabetes mellitus, obstructive sleep apnea, high blood pressure, cardiovascular disease, some types of neoplasms (endometrium, breast, and liver), and degenerative joint disease is well known. These complications vary widely among obese subjects. A subgroup of obese individuals, termed “metabolically healthy obese” (MHO), has a favorable metabolic profile characterized by high insulin sensitivity, low visceral adipose tissue content, less liver fat, normal blood pressure, and favorable lipid, inflammatory, hormonal, and immunological profiles despite having excessive body fat [1]. MHO is an emerging phenotype with disease risks that are somewhere between those of healthy, normal-weight individuals, and unhealthy, obese individuals. In general, these subjects have a lower risk of diabetes and cardiovascular disease than obese individuals with metabolic abnormalities [2]. In regard to diabetes, 1 study found that the development of cardiometabolic abnormalities in one-third of the MHO subjects studied led to a significantly increased risk of diabetes. In regard to cardiovascular diseases (CVD), studies have found that MHO subjects have CVD risks similar to those of normal-weight subjects. Recently, the benign nature of the MHO phenotype in the long term has begun to be questioned [3].

Adipose tissue used to be considered as simply an energy deposit. However, nowadays, the function of adipose tissue in metabolism as an endocrine organ responsible for the secretion of bioactive molecules—adipokines—is known [4]. Adipokines contribute to the inflammatory process, which can lead to obesity-associated cardiometabolic complications. MHO individuals seem to be protected from or are more resistant to developing these complications. The mechanisms that may explain the favorable metabolic profile of these individuals are still unknown, and it is intriguing why some MHO individuals develop comorbidities and others do not. Characteristics of adipose tissue, such as the proinflammatory profile and expression of adipokines, may be involved [1]. Adipokines have a hormonal function, act as growth factors that modulate insulin resistance, influence fat and glucose metabolism, and play a role in pro- and anti-inflammatory responses. The different types of adipokines, such as adiponectin and resistin, are the most studied [5]. Furthermore, inflammatory biomarkers such as interleukin 6 (IL-6) [6], tumor necrosis factor-alpha (TNFa), and C-reactive protein (CRP) decrease with dietary intervention and weight loss [7].

Few studies have analyzed the metabolic effects of lifestyle interventions (LSI) involving a restrictive diet and/or exercise in MHO subjects. Studies that have been done show contradictory results [8]. Principally, prior studies have shown that combining a Mediterranean diet with moderate-to-high-intensity aerobic training is effective at improving body composition [9], but no studies have analyzed if adipokine levels and the inflammatory profile experience significant variations when MHO subjects undergo intensive lifestyle modifications. Therefore, our principal objective was to evaluate whether changes in adipokine and inflammatory biomarker levels observed after 2 years of a personalized intervention were related to a lifestyle modification program based on the Mediterranean diet and physical exercise.

## 2. Materials and Methods

### 2.1. Subjects and Study Design

An open-label, nonrandomized, interventional, and analytical study was performed on a population of MHO women to determine the effectiveness in 2 years of a weight loss intervention (≥5% of body weight). We conducted a study on a population of 115 MHO women belonging to 4 health centers in the Malaga District of the Andalusian Health Service. Participants were recruited by their general practitioners between June 2013 and April 2014. After obtaining written informed consent, the subjects underwent a clinical interview with a doctor of internal medicine, a nurse, and a nutritionist at the Regional University Hospital of Malaga (the Civil Hospital); these practitioners were their contact person during the study.

We followed the methods of Rodriguez-Garcia et al. [10]. The participants were considered to be MHO if they met ≤1 of these 4 metabolic syndrome criteria: fasting plasma glucose ≥ 5.5 mmol/l, blood pressure ≥ 135/85 mmHg (or use of blood pressure-lowering agents), HDL-cholesterol ≤ 1.30 mmol/l, or triglycerides ≥ 1.70 mmol/l (or use of lipid-lowering therapies).

### 2.2. Materials

All samples were managed and stored at IBIMA's Hospital Biobank of Malaga, which belongs to the Andalusian Public Health System Biobank, part of the Spanish National Biobank Network (project PT13/0010/0006). The informed consent form and protocols were approved by the institutional ethics committee (Comité Coordinador de Ética de la Investigación Biomédica de Andalucía). This study is listed on the ISRCTN registry with trial ID ISRCTN88315555.

The population underwent an intervention involving a hypocaloric Mediterranean diet and a physical exercise program. In addition, the participants filled out a validated food frequency questionnaire [11]. Adherence to the Mediterranean diet was measured using a validated questionnaire, as described by Trichopoulou et al. [12]. Patients were considered to have dropped out if there was lack of adherence to the scheduled visits with specialists from the study.

The physical activity program recommended daily exercise. To verify that participants met the proposed physical exercise goal, they were monitored using a pedometer (On Step PE12) and they were evaluated using the Rapid Assessment of Physical Activity (RAPA) questionnaire [13], a validated 7-item questionnaire. Sedentarism or light physical activity corresponded to a score of 1-3 points, moderate to a score of 4-5 points, and vigorous to a score of 6–7 points. In total, over the course of the study, participants completed 3 different questionnaires: 2 concerned nutrition and 1 concerned physical exercise. These 3 questionnaires were completed on 4 occasions (baseline, 3 months, 1st year, and 2nd year).

After 2 years of follow-up, the subjects were classified into 3 groups based on the percentage of weight loss with respect to their baseline body weight: <5%, ≥5% to <10%, and ≥10%.

### 2.3. Methods

40 ml of peripheral blood was collected from each participant at 4 different periods (baseline, 3 months, 1st year, and 2nd year). Vacutainer® ethylenediaminetetraacetic acid (EDTA) spray-coated tubes were used for whole blood hematology measurements, and Vacutainer® Plus plastic serum tubes were used for serum determinations. These tubes were connected to a Vacutainer® Push Button Blood Collection Set and a tube and syringe holder for blood collection and for producing a vacuum inside the tube. They were placed on ice. The samples were immediately centrifuged at 2772 ×g for 15 minutes at 4°C (plasma) or at room temperature (serum). Plasma and serum were aliquoted and stored at -80°C until analysis.

In the Clinical Analysis Department of the Regional University Hospital of Malaga, blood glucose was determined by the glucose oxidase method adapted to an autoanalyzer (Dimension®, Dade Behring, Germany) and HbA1c (%) was measured by high-performance liquid chromatography (HPLC). For the lipid profile, total cholesterol and triglycerides were measured by enzymatic methods using the commercial equipment (Dimension®, Dade Behring, Germany). High-density lipoprotein-linked cholesterol (HDL-c) and low-density lipoprotein-linked cholesterol (LDL-c) were both measured by homogeneous direct measurement methods that do not require any preliminary treatment of the sample.

In the Research Laboratory of IBIMA, adipokines and inflammatory parameters were measured. Serum adipokine and inflammatory biomarker levels (IL-6 and TNFa) were measured using an enzyme-linked immunosorbent assay (ELISA) (R&D Systems Inc., Minneapolis, MN, USA). For adiponectin levels, the minimum detectable concentration was 0.246 ng/ml. The intra- and interassay coefficients of variation were 3.5% and 6.5%, respectively. For resistin levels, the minimum detectable concentration was 0.026 ng/ml. The intra- and interassay coefficients of variation were 4.7% and 8.4%, respectively. For IL-6 levels, the minimum detectable concentration was 0.70 pg/ml. The intra- and interassay coefficients of variation were 2.6% and 4.5%, respectively. Lastly, for TNFa levels, the minimum detectable concentration was 1.6 pg/ml. The intra- and interassay coefficients of variation were 4.7% and 5.8%, respectively.

High-sensitivity CRP levels were measured using ELISA (DRG Instruments GmbH, Germany). The minimum detectable concentration was 0.1 mg/ml. The intra- and interassay coefficients of variation were 4.4% and 3.3%, respectively.

### 2.4. Statistical Analysis

We based our calculations to calculate the sample size on the previous MHO study [8]. A population sample of 115 MHO women was required.

Relationships between serum levels of adipokines and inflammatory biomarkers and adherence to the Mediterranean diet and physical exercise were examined by means of the Pearson correlation analysis. Quantitative variables were analyzed as mean ± standard deviation (SD), and qualitative variables were expressed as percentages. Student's *t*-test, one-way analysis of variance (ANOVA) test, and post hoc analysis were used to compare quantitative variables whereas the Chi-squared test, the Mantel-Haenszel test, and the Mann-Whitney test were used for nonparametric variables.

We used the SPSS® statistical program for Windows version 22.0 (IBM Corporation Inc., Somers, NY, USA) to analyze the results.

## 3. Results

This study began with 115 women. After 2 years of a hypocaloric diet (Mediterranean diet) and physical exercise, there were 55 dropouts (52.0%). Thus, the final sample included 67 women. They were classified into 3 groups according to the percentage of weight loss with respect to their baseline body weight: Group 1: <5% (*n* = 23), Group 2: ≥5% to <10% (*n* = 22), and Group 3: ≥10% (*n* = 22) (*p* = 0.272). The mean (±SD) age of the women was 44.5 (±3.6 years), with no significant differences between the 3 groups (*p* = 0.389).

After 2 years of intensive intervention in the population as a whole, we found a decrease in parameters, including body weight, BMI, and waist circumference (all *p* < 0.0001), with respect to baseline conditions. However, no statistically significant differences were found in blood pressure or in analytical parameters such as the glycemic profile, HbA1c, or the lipid profile, though these values did tend to decline. All analytical parameter mean values were within normal ranges at baseline.

According to the Kaplan-Meier estimator, the number of patients who reached the goal rate (weight loss ≥ 5%) was 67 participants (58.3%) during the intervention program (24 months). Of these, 57 participants (54.6%) reached the goal weight during the first 3 months and 67 participants (58.3%) reached the goal weight in the first year.

When analyzing the 3 weight loss groups individually after the intervention, significant differences were found between them. Differences were seen principally between Group 2 and Group 3 in regard to body weight (*p* = 0.003 and *p* < 0.001, respectively), BMI (*p* = 0.005 and *p* < 0.001, respectively), and waist circumference (*p* = 0.002 and *p* > 0.001, respectively) although all groups decreased their anthropometric parameters with respect to baseline conditions. However, parameters such as blood pressure, glucose, HbA1c, or the lipid profile did not show significant differences. Only glucose levels in Group 1 (*p* = 0.012) and triglyceride levels in Group 2 (*p* = 0.003) were significantly reduced.  Table 1 shows changes in anthropometric and analytical parameters at baseline and after 2 years of intervention, according to the percentage of weight loss.

A positive association was found between adherence to the Mediterranean diet and amount of weight loss. At baseline, all groups showed moderate adherence to the Mediterranean diet, without significant differences between them (*p* = 0.132). After 2 years of intervention, adherence varied in accordance with the level of weight loss (*p* = 0.004, between Groups 1 and 3).  Table 2 summarizes adherence to Mediterranean diet according to weight loss.

At the beginning of the study, participants had light (62 participants (53.6%)), moderate (35 participants (30.4%)), and vigorous (18 participants (16.1%)) levels of physical activity. After 2 years of training with a monitor, these levels of physical activity changed: 11 of the participants (16.1%) had light activity, 29 (42.9%) had moderate activity, and 27 (41.1%) had vigorous activity levels. This physical activity of the different weight loss groups during the study is summarized in Table 3.

The adipokine profile for these MHO women is summarized in Table 4. For the entire population, adiponectin levels decreased after the intervention (*p* = 0.039). When comparing the different weight loss groups, only Group 1 showed a significant reduction in adiponectin levels (*p* = 0.003). However, Group 3, the group which lost the most weight, saw an increase in adiponectin levels, though the difference was not statistically significant (*p* = 0.911).

Resistin levels increased significantly in the entire study population following the intervention (*p* < 0.001). However, when analyzing each group, only the increases in serum resistin levels in Group 1 (*p* < 0.001) and Group 3 (*p* < 0.001) were statistically significant.

There were significant decreases in hsCRP, IL-6, and TNFa levels in the entire population following the intervention (*p* < 0.001 for all parameters). Serum hsCRP levels decreased in all groups (Group 1: *p* = 0.003, Group 2: *p* = 0.010, and Group 3: *p* = 0.097). Serum IL-6 levels also decreased in all groups (Group 1: *p* < 0.001, Group 2: *p* = 0.001, and Group 3: *p* < 0.001), and serum TNFa levels decreased in Group 1 (*p* = 0.001) and Group 3 (*p* < 0.001), but increased slightly in Group 2 (*p* = 0.019).

In this study, our aim was to verify whether different adipokines (adiponectin and resistin) and inflammatory biomarkers (CRP, IL-6, and TNFa) correlated with adherence to Mediterranean diet and physical exercise in our MHO population. Upon analyzing adipokines and inflammatory biomarkers, it was found that only CRP and adiponectin were shown to be correlated with adherence to the Mediterranean diet after 2 years of intervention. CRP showed a negative correlation (*r* = ‐0.292, *p* = 0.030), and adiponectin showed a positive correlation (*r* = 0.340, *p* = 0.011). Moreover, as expected, physical exercise was shown to be correlated with adherence to the Mediterranean diet after 2 years of intervention (*r* = 0.414, *p* = 0.002).

## 4. Discussion

Our study focuses on analyzing adipokine and inflammatory biomarker profiles involved in the pathogenesis of inflammation found in metabolically healthy obese individuals after 2 years of lifestyle modification based on a calorie-restricted Mediterranean diet and physical exercise. We provide evidence that clinically significant weight loss in MHO women leads to limited improvement in serum levels of adipokines and inflammatory biomarkers. We provide further evidence for the concept of the MHO phenotype as a subgroup that is resistant to the metabolic disorders associated with obesity and that weight loss does not substantially improve this phenotype's metabolic profile.

The intervention in this study produced different effects on energy metabolism and improved health parameters, such as BMI, in the MHO subjects evaluated. In this study, the weight loss resulting from the Mediterranean diet and exercise had effects on all metabolic parameters after the intervention. Though some results were not statistically significant (glucose and lipid profile), changes in the levels of these parameters may be clinically relevant in the management of future obesity-related pathologies.

In the adipokine profile, adiponectin has a wide spectrum of metabolic and anti-inflammatory effects by its inhibition of monocyte adhesion to endothelial cells, macrophage transformation to foam cells, and endothelial cell activation. We found a significant decrease in serum adiponectin levels in the study population as a whole, though they remained within normal range. These data were in accordance with Rondanelli et al. [14]. However, in adiponectin levels measured according to the different weight loss groups, we found that this significant difference was not noted in groups with a greater weight loss. In fact, in the group with ≥10% of weight loss, serum adiponectin levels increased. This group had a high adherence to the Mediterranean diet and a vigorous physical exercise score after 2 years of intervention. It has been demonstrated that exercise promotes a decrease in the body mass and body mass index along with an increase in insulin sensitivity and adiponectin concentration [15]. Adiponectin is mostly expressed in subcutaneous adipose tissue. Its expression and blood concentration decrease as adiposity increases. However, some authors have shown that individuals with higher subcutaneous rather than visceral adipose tissue distribution have higher levels of adiponectin. It is not known whether MHO subjects have predominantly peripheral adiposity along with higher adiponectin levels [16]. MHO women have an adipokine profile somewhere between at-risk obese and metabolically benign normal-weight women and have higher adiponectin levels than at-risk obese women [17]. Although adiponectin levels are lower in obese subjects, MHO subjects might present a paradoxical hyperadiponectinemia that could be a clue in explaining their favorable metabolic profile.

Adiponectin binds to its AdipoR1 or AdipoR2 receptors in muscle, liver, and adipose tissue, increasing the activity of adenosine monophosphate kinase (AMPK) or peroxisome proliferator-activated receptor- (PPAR-) *α*. Reducing adipose tissue mass, through weight loss in association with physical exercise, can increase adiponectin concentrations as occurred in our participants who lose more than 10% of their body weight. On the other hand, the effect of TNFa on obesity increased the release of fatty acid by adipocytes, reduced adiponectin synthesis, and impaired insulin signalling [18].

It has also been shown that the adipose tissue of MHO individuals has a favorable inflammatory profile associated with high levels of adiponectin [19]. In contrast, in other studies, no differences in adiponectin levels among MHO individuals were found [20].

The role of resistin in human obesity is unclear. There are few studies on the profile of resistin in the MHO, though studies have shown that the level of resistin does not significantly change between metabolic phenotypes [17]. However, other studies have shown that the MHO phenotype is characterized by low levels of resistin. Our results showed a significant increase in resistin levels in both the total study population and in the groups of participants who had moderate adherence to the Mediterranean diet and moderate-vigorous physical exercise, data in concordance with findings of Gómez-Ambrosi et al. [21]. The effects of resistin may be mediated by the paracrine and endocrine ways, probably via binding of resistin to a surface receptor on target cells. Resistin can activate cytokines such as CRP, IL-6, IL-12, and TNF via the NF*κ*B pathway [22].

Obesity as well as BMI and waist circumference are associated with CRP levels [23]. Postmenopausal women with the MHO phenotype have lower levels of CRP. It has been theorized that a lower-grade state of inflammation could play a role in the protective metabolic profile of the MHO individuals [24]. Evidence from interventional studies has demonstrated that weight loss by caloric restriction reduces several inflammatory biomarkers, including CRP, IL-6, and TNFa [25]. Our population saw a significant reduction in CRP levels, a finding that was independent of the percentage of weight loss but which was significantly associated with a decrease in waist circumference. These results are in accordance with the findings of Nakamura et al. [26].

IL-6 is an inflammatory biomarker that is positively correlated with obesity, glucose intolerance, and insulin resistance [27]. The production of IL-6 is 3 times higher in visceral adipose tissue than in subcutaneous adipose tissue [27], which could explain the decrease in IL-6 levels in MHO women after weight loss. Both adipocyte hypertrophy and inflammatory stimuli, such as TNF, favor an increase in IL-6. It has been reported that weight loss reduces both circulating levels and adipose tissue expression of IL-6 [4]. Similar IL-6 levels in lean and MHO individuals have been found [28], data that are in agreement with those found in our study, as our population maintained normal levels of both IL-6 and TNFa. MHO subjects have lower levels of TNFa and a reduced proinflammatory profile in comparison with other obese phenotypes [18]. TNF is also involved in the synthesis of proinflammatory cytokines, such as IL-6 and TNF itself, through NF*κ*B activation. TNF is a factor involved in diet-induced obesity and insulin resistance as well as in low-grade inflammation related to adipose tissue expansion. We found improved CRP, IL-6, and TNFa serum levels after dietary intervention in both the total population and in the different weight loss groups, data in accordance with Poelkens et al. [29].

Studies assessing the short-term effect of the Mediterranean diet [30] and exercise [31] in obese subjects have shown reductions in inflammatory parameters. However, in contrast with other authors [8], we did not find that weight loss improved the profile of several inflammatory markers (CRP, IL-6, and TNFa) in subjects who lost ≥10% of their baseline body weight.

## 5. Limitations

Our study has some limitations. Our population comprised middle-aged Caucasian women; thus, we cannot extrapolate our conclusions to MHO patients of other races, sexes, or ages. Another important limitation was the losses during follow-up, perhaps due to participants growing tired of the restrictive diet.

Although public health messaging should continue to promote healthy lifestyle habits for all obese patients, the debatable results of lifestyle modifications in MHO individuals would justify, for reasons of cost efficiency, prioritizing these types of intensive interventions in metabolically abnormal obese individuals and monitoring MHO subjects for early detection of the development of metabolic abnormalities.

## 6. Conclusion

Weight loss reduces inflammatory biomarkers in the MHO but induces a deterioration in the adipokine profile, which does not improve with diet and exercise intervention. These findings allow us to clarify mechanisms behind inflammation and metabolic disorder genesis so as to prevent the development of obesity-associated comorbidities.

## Figures and Tables

**Table 1:** Parameters at baseline (*n* = 115 MHO participants) vs. after 2 years (*n* = 67 MHO participants) of lifestyle modification, according to percentage of weight loss.

	Weight loss group	Baseline	2 years	*p*
Age (years)	**<5%**	43.7 ± 3.4	—	
	**≥5-<10%**	45.0 ± 2.4	—	
	**≥10%**	45.4 ± 4.5	—	
	**All**	44.5 ± 3.6	46.5 ± 3.6	

Body weight (kg)	**<5%**	91.2 ± 13.8	86.7 ± 10.3	0.698
	**≥5-<10%**	95.0 ± 15.8	86.3 ± 17.8	0.003
	**≥10%**	90.8 ± 12.4	79.1 ± 12.4	<0.001
	**All**	92.7 ± 13.8	83.8 ± 13.4	<0.001

BMI (kg/m^2^)	**<5%**	35.5 ± 3.6	34.3 ± 3.2	0.724
	**≥5-<10%**	37.2 ± 5.8	34.4 ± 6.5	0.005
	**≥10%**	36.0 ± 4.5	31.5 ± 4.6	<0.001
	**All**	36.3 ± 4.7	33.3 ± 4.8	<0.001

Waist circumference (cm)	**<5%**	111.6 ± 10.8	108.1 ± 9.5	0.831
	**≥5-<10%**	116.4 ± 11.4	108.8 ± 15.5	0.002
	**≥10%**	114.7 ± 11.2	100.9 ± 13.1	<0.001
	**All**	111.7 ± 11.1	105.6 ± 12.8	<0.001

SBP^∗^/DBP^∗∗^ (mmHg)	**<5%**	113 ± 15/75 ± 9	114 ± 14/73 ± 12	0.285/0.302
	**≥5-<10%**	115±10/78±7	115 ± 12/76 ± 11	0.501/0.342
	**≥10%**	110±14/76±11	118 ± 20/74 ± 11	0.498/0.402
	**All**	114 ± 14/76 ± 9	116 ± 16/74 ± 11	0.532/0.199

Glucose (mmol/l)	**<5%**	4.89 ± 0.40	4.72 ± 0.43	0.012
	**≥5-<10%**	4.86 ± 0.54	4.77 ± 0.41	0.833
	**≥10%**	4.74 ± 0.44	4.62 ± 0.36	0.376
	**All**	4.85 ± 0.44	4.76 ± 0.39	0.214

HbA1c (%)	**<5%**	5.3 ± 0.2	5.4 ± 0.3	0.635
	**≥5-<10%**	5.4 ± 0.3	5.4 ± 0.3	0.333
	**≥10%**	5.3 ± 0.3	5.3 ± 0.3	0.801
	**All**	5.4 ± 0.3	5.4 ± 0.3	0.645

Total cholesterol (mmol/l)	**<5%**	5.05 ± 0.765	5.08 ± 0.80	0.578
	**≥5-<10%**	4.89±0.77	4.81 ± 0.62	0.098
	**≥10%**	5.15 ± 0.71	4.97 ± 0.82	0.478
	**All**	5.04 ± 0.73	4.98 ± 0.76	0.941

LDL cholesterol (mmol/l)	**<5%**	3.13 ± 0.69	3.17 ± 0.76	0.445
	**≥5-<10%**	2.89 ± 0.63	2.92 ± 0.56	0.902
	**≥10%**	3.08 ± 0.79	3.04 ± 0.66	0.789
	**All**	3.05 ± 0.69	3.06 ± 0.67	0.501

HDL cholesterol (mmol/l)	**<5%**	1.41 ± 0.32	1.37 ± 0.21	0.112
	**≥5-<10%**	1.53 ± 0.28	1.53 ± 0.22	0.584
	**≥10%**	1.54 ± 0.32	1.54 ± 0.31	0.897
	**All**	1.48 ± 0.31	1.47 ± 0.26	0.632

Triglycerides (mmol/l)	**<5%**	0.98 (0.76–1.29)	0.98 (0.77–1.47)	0.545
	**≥5-<10%**	0.88 (0.66–1.39)	0.70 (0.62–0.99)	0.003
	**≥10%**	0.93 (0.69–1.18)	0.85 (0.72–1.08)	0.497
	**All**	0.95 (0.75–1.27)	0.89 (0.70–1.23)	0.132

Group 1: weight loss <5% (baseline, *n* = 47; 2^nd^ year, *n* = 23); Group 2: weight loss ≥5-<10% (baseline, *n* = 27; 2^nd^ year, *n* = 22); Group 3: weight loss ≥10% (baseline, *n* = 30; 2^nd^ year, *n* = 22). ^∗^SBP: systolic blood pressure; ^∗∗^DBP: diastolic blood pressure. Normal values (NV): glucose NV: 3.89-6.16; HbA1c NV: 4.0-6.0; total cholesterol NV: <5.18; LDL cholesterol NV: <3.37; HDL cholesterol NV: >1.30; triglycerides NV: <1.70.

**Table 2:** Adherence to Mediterranean diet at baseline (*n* = 115 MHO participants) vs. after 2 years (*n* = 67 MHO participants) of lifestyle modification, according to percentage of weight loss.

	Weight loss group	Baseline	2 years	*p*
Very low adherence (*n* (%))	**<5%**	3 (6.4)	0 (0.0)	—
	**≥5-<10%**	2 (7.4)	0 (0.0)	—
	**≥10%**	4 (13.3)	0 (0.0)	—
	**All**	9 (8.0)	0 (0.0)	—

Low adherence (*n* (%))	**<5%**	16 (34.0)	6 (27.3)	0.006
	**≥5-<10%**	13 (48.1)	0 (0.0)	—
	**≥10%**	11 (36.7)	1 (5.0)	<0.001
	**All**	40 (41.6)	7 (12.7)	0.001

Moderate adherence (*n* (%))	**<5%**	28 (59.6)	14 (63.6)	0.01
	**≥5-<10%**	12 (44.4)	12 (92.3)	0.006
	**≥10%**	15 (50.0)	10 (50.0)	0.824
	**All**	55 (50.4)	36 (65.5)	0.003

High adherence (*n* (%))	**<5%**	0 (0.0)	2 (9.1)	—
	**≥5-<10%**	0 (0.0)	1 (7.7)	—
	**≥10%**	0 (0.0)	9 (45.0)	—
	**All**	0 (0.0)	12 (21.8)	—

Group 1: weight loss <5% (baseline, *n* = 47; 2^nd^ year, *n* = 23); Group 2: weight loss ≥5-<10% (baseline, *n* = 27; 2^nd^ year, *n* = 22); Group 3: weight loss ≥10% (baseline, *n* = 30; 2^nd^ year, *n* = 22). Adherence to Mediterranean diet was measured as follows: very low adherence <5 points, low adherence ≥5-<8 points, moderate adherence ≥8-<12 points, and high adherence ≥12 points.

**Table 3:** Physical activity levels at baseline (*n* = 115 MHO participants) vs. after 2 years (*n* = 67 MHO participants) of lifestyle modification, according to percentage of weight loss.

	Weight loss group	Baseline	2 years	*p*
Sedentarism or light level (*n* (%))	**<5%**	23 (50.0)	5 (22.7)	0.01
	**≥5-<10%**	15 (55.6)	3 (23.1)	0.002
	**≥10%**	17 (56.7)	1 (4.8)	<0.001
	**All**	55 (53.5)	9 (16.0)	0.001

Moderate level (*n* (%))	**<5%**	16 (34.8)	12 (54.5)	0.006
	**≥5-<10%**	8 (29.6)	5 (38.5)	0.068
	**≥10%**	6 (20.0)	7 (33.3)	0.010
	**All**	30 (30.4)	24 (42.9)	0.002

Vigorous level (*n* (%))	**<5%**	7 (15.2)	5 (22.7)	0.01
	**≥5-<10%**	4 (14.8)	5 (38.5)	0.006
	**≥10%**	7 (23.3)	13 (61.9)	<0.001
	**All**	18 (16.1)	23 (41.1)	0.003

Group 1: weight loss <5% (baseline, *n* = 47; 2^nd^ year, *n* = 23); Group 2: weight loss ≥5-<10% (baseline, *n* = 27; 2^nd^ year, *n* = 22); Group 3: weight loss ≥10% (baseline, *n* = 30; 2^nd^ year, *n* = 22). Physical activity levels according to the Rapid Assessment of Physical Activity (RAPA) questionnaire are as follows: sedentarism or light level RAPA = 1‐3 points, moderate level RAPA = 4‐5 points, and vigorous level RAPA = 6‐7 points.

**Table 4:** Adipokine and inflammatory biomarker levels at baseline vs. after 2 years of lifestyle modification, according to percentage of weight loss. MHO participants: baseline, *n* = 115; 3 months, *n* = 104; 1^st^ year, *n* = 75; and 2^nd^ year, *n* = 67.

	Weight loss	Baseline	3 months	1 year	2 years	*p* (baseline vs. 2 y)
Adiponectin (ng/ml)	**<5%**	7.0 ± 2.1	7.1 ± 2.5	7.7 ± 3.5	5.8 ± 2.4	0.003
	**≥5-<10%**	7.8 ± 3.0	9.0 ± 4.3	12.6 ± 5.1	6.7 ± 3.1	0.699
	**≥10%**	7.7 ± 3.1	7.4 ± 3.4	12.6 ± 5.5	8.6 ± 4.8	0.911
	**All**	7.4 ± 2.7	7.7 ± 3.4	10.7 ± 5.2	7.05 ± 3.7	0.039

Resistin (ng/ml)	**<5%**	5.5 ± 2.4	4.6 ± 1.2	6.1 ± 2.8	7.9 ± 3.4	<0.001
	**≥5-<10%**	5.2 ± 2.2	5.2 ± 1.6	5.5 ± 1.9	7.5 ± 4.4	0.08
	**≥10%**	5.2 ± 1.8	5.8 ± 2.3	5.4 ± 1.9	8.8 ± 4.3	<0.001
	**All**	5.3 ± 2.2	5.1 ± 1.8	5.7 ± 2.4	8.1 ± 2.9	<0.001

hsCRP (mg/l)	**<5%**	5.4 ± 2.9	5.0 ± 2.7	4.1 ± 2.1	3.3 ± 3.1	0.003
	**≥5-<10%**	5.2 ± 2.5	4.6 ± 2.4	3.4 ± 2.4	3.2 ± 2.8	0.010
	**≥10%**	5.2 ± 2.6	4.4 ± 2.3	3.8 ± 3.04	3.2 ± 3.3	0.097
	**All**	5.3 ± 2.7	4.7 ± 2.5	3.8 ± 2.5	3.3 ± 3.1	<0.001

IL-6 (pg/ml)	**<5%**	4.9 ± 0.9	4.8 ± 1.2	7.4 ± 1.9	2.5 ± 0.7	<0.001
	**≥5-<10%**	5.1 ± 1.2	6.0 ± 2.6	6.9 ± 2.0	2.5 ± 0.4	0.001
	**≥10%**	4.5 ± 0.8	5.5 ± 2.7	7.5 ± 3.2	2.4 ± 0.7	<0.001
	**All**	4.8 ± 1.0	5.3 ± 2.2	7.3 ± 2.5	2.4 ± 0.6	<0.001

TNFa (pg/ml)	**<5%**	13.3 ± 1.0	12.5 ± 1.4	11.3 ± 2.7	10.9 ± 3.4	0.001
	**≥5-<10%**	13.6 ± 0.6	11.8 ± 2.3	11.4 ± 2.1	13.8 ± 1.6	0.019
	**≥10%**	13.9 ± 2.5	12.7 ± 2.7	10.4 ± 2.3	9.8 ± 0.8	<0.001
	**All**	13.6 ± 1.5	12.4 ± 2.1	11.0 ± 2.5	11.1 ± 2.9	<0.001

Group 1: weight loss <5% (baseline: 3 months, *n* = 47; 1^st^ year, *n* = 20; and 2^nd^ year, *n* = 23); Group 2: weight loss ≥5-<10% (baseline: 3 months, *n* = 27; 1^st^ year, *n* = 27; and 2^nd^ year, *n* = 22); Group 3: weight loss ≥10% (baseline: 3 months, *n* = 30; 1^st^ year, *n* = 28; and 2^nd^ year, *n* = 22). Normal values (NV): adiponectin NV: 0.8–21; resistin NV: 6.1–26.4; hsCRP NV: 0.07-8.2; IL-6 NV: 3.13–12.5; TNFa NV: <15.6.

## Data Availability

Data availability will be provided previous requeriment to the corresponding authors.
